# Physiological and metabolomic analyses reveal the mechanism by which exogenous spermine improves drought resistance in alfalfa leaves (*Medicago sativa* L.)

**DOI:** 10.3389/fpls.2024.1466493

**Published:** 2024-10-09

**Authors:** Wenjuan Wang, Wenjuan Kang, Shangli Shi, Linbo Liu

**Affiliations:** Key Laboratory of Grassland Ecosystem (Gansu Agricultural University), Ministry of Education, Lanzhou, China

**Keywords:** alfalfa, exogenous, spermine, physiological, metabolomics, drought resistance

## Abstract

**Introduction:**

Alfalfa (*Medicago sativa* L.) is a globally important legume crop with high nutritional and ecological value. Drought poses a serious threat to alfalfa acreage and yields. Spermine (Spm) has been shown to protect plants from drought damage. The aim of this study was to clarify the mechanism of exogenous Spm to improve drought resistance of alfalfa.

**Methods:**

In this study, we root applied 0.1, 0.5, and 1 mM Spm to Gannong No. 3 (G3) alfalfa under drought stress, and then determined their physiological and metabolic changes.

**Results:**

The results showed that exogenous Spm increased chlorophyll content, chlorophyll fluorescence parameters and gas exchange parameters, enhanced antioxidant enzymes activity, improved ascorbic acid-glutathione (AsA-GSH) cycle, increased osmoregulatory substances content, reduced hydrogen peroxide and superoxide anion levels, and inhibited malondialdehyde accumulation in alfalfa under drought stress, thereby increasing plant height and leaf relative water content and enhancing drought tolerance of alfalfa. The redundancy analysis of the above physiological indicators showed that the addition of the optimal Spm to improve drought tolerance of alfalfa under drought stress was mainly achieved by increasing catalase activity and improving the ASA-GSH cycle. In addition, metabolomics analysis revealed that exogenous Spm increased the content of oxobutanedioic acid, citric acid, fumaric acid and malic acid to enhance the tricarboxylic acid cycle. Meanwhile, exogenous Spm increased endogenous Spm and proline (Pro) content to resist drought stress by enhancing Spm and Pro metabolism. Moreover, exogenous Spm increased the accumulation of the signaling substance abscisic acid.

**Discussion:**

In conclusion, exogenous Spm enhanced drought resistance of alfalfa leaves under drought stress.

## 1 Introduction

On December 1, 2023, the Secretariat of the United Nations Convention to Combat Desertification (UNCCD) released the Global Drought Profile 2023 report, stating that drought on a global scale has reached an “unprecedented state of emergency” (United Nations News, 2023). Drought, as one of the major abiotic stressors, severely inhibits crop distribution, growth, and yield (Razi and Muneer, 2021). In recent decades, global warming has led to numerous weather events and severe droughts (Qiu et al., 2023). It is predicted that by 2050, 50% of the arable land needed for crops will be negatively affected by drought (Kumar et al., 2024). During drought, the metabolic rate of plants tends to decrease, affecting their development and growth. Drought stress induces complex physiological and biochemical responses in plant cells by disturbing water homeostasis and reducing plant water utilization efficiency. Excessive drought can lead to irreversible cell damage and ultimately cell death in plants. Alfalfa (*Medicago sativa* L.) is an essential perennial legume that is grown worldwide. However, alfalfa growth is largely limited by drought (Liu et al., 2023).

Polyamines (PAs) are low molecular weight aliphatic nitrogen-containing bases produced during biological metabolism. The three primary PAs in plants are putrescine (Put), spermidine (Spd) and spermine (Spm). PAs are involved in plant defense against various abiotic and biotic threats (Hussain et al., 2011). Numerous studies have shown that PAs play an important role in defense against abiotic stresses in legumes. Exogenous Spd enhanced the heat tolerance of white clover by strengthening the antioxidant system and slowing down the degradation of chlorophyll (Chl) (Luo et al., 2020). Exogenous Spd improved the activities of catalase (CAT), superoxide dismutase (SOD), ascorbate peroxidase (APX) and guaiacol peroxidase to alleviate the negative effects of drought stress on faba bean seedlings (Abid et al., 2022). Spm pretreatment increased ascorbic acid (ASA)- glutathione (GSH) cycle and reduced reactive oxygen species (ROS) and malondialdehyde (MDA) in mung bean plants under drought stress (Nahar et al., 2017). Exogenous application of Spm significantly improved SOD, CAT, peroxidase (POD), glutathione reductase (GR), APX activities and reduced MDA in common bean seedlings under arsenic stress (Shah et al., 2022). Moreover, exogenous PAs improved the water status by increasing the levels of proline (Pro), soluble protein (SP) and soluble sugar (SS), which improved the drought resistance of mung bean plants (Sadeghipour, 2019).

Plants adjust physiological, biochemical and phenotypic responses on the molecular, enzymatic and metabolic levels to adapt to stressful conditions (Li et al., 2021). In recent years, increasing attention has been paid to the role of metabolites in stress tolerance, but the analysis and characterization of stress-responsive genes and proteins in plant species is still the mainstay (Ghatak et al., 2018). Metabolomics has been used to reveal changes in metabolites associated with drought, heat and salt tolerance in plants. It has been demonstrated that exogenous melatonin application reduces the accumulation of flavonoids and increases the accumulation of amino acids and their derivatives (for example, L-asparagine, DL-homocysteine, L-phenylalanine, L-(-)-tyrosine), which enhance plant growth and yield in soybean plants subjected to drought stress (Zou et al., 2021). Metabolomic analysis revealed that Spm application increased γ-aminobutyric acid, glutamate and alanine, and tricarboxylic acid (TCA) cycle intermediate metabolites (aconitic acid) in creeping bentgrass exposed to salt stress (Li et al., 2022). Furthermore, metabolomics results revealed that wheat treated with trehalose exhibited increased levels of protective metabolites such as amino acids, purines, phenylpropanoids and flavonoids under heat stress (Luo et al., 2022).

However, little is known about whether exogenous PA application can positively regulate drought tolerance in alfalfa, which physiological indicators can be increased or decreased by exogenous PA application to regulate drought tolerance in alfalfa, and which metabolic pathways are utilized to improve drought resistance of alfalfa. Our previous study revealed that low content of Put and high content of Spd and Spm in alfalfa are beneficial to the resistance to drought stress (Wang et al., 2023). This may be because high-molecular-weight PAs such as Spd and Spm contain one or two, respectively, more amino groups than low-molecular-weight PAs (Put) (Liu et al., 2015). This results in Spm carrying more nitrogen, the adequate supply of which is essential for plant growth, development and stress response (Moschou et al., 2012). In addition, a review showed that Spm plays an important role in improving drought tolerance in plants (Hasan et al., 2021). In this study, we focused on the effect of exogenous Spm (the highest molecular weight PA) on drought tolerance of alfalfa under drought stress. The objectives of this study were 1) to investigate the physiological response (including photosynthesis, osmoregulation and antioxidant system) of alfalfa leaves to exogenous Spm under drought stress; 2) to explore the effects of exogenous Spm on metabolites of alfalfa under drought stress.

In this study, we focused on the effects of exogenously applied Spm (the highest molecular weight PA) on physiological responses, including photosynthesis, osmoregulation, and oxidative damage, as well as changes in organic metabolites based on metabolomics in alfalfa leaves under drought stress. The present findings will help to better understand the critical role of Spm and its metabolites in the alfalfa response to drought stress, provide a theoretical basis for exogenous Spm to enhance drought resistance in alfalfa, and offer insight for exogenous Spm application in the production of stress-tolerant alfalfa.

## 2 Materials and methods

### 2.1 Materials and experimental design

The experiment was conducted in the Key Laboratory of Grassland Ecosystem of the Ministry of Education. Spm and PEG-6000 were purchased from Beijing Solarbio Science and Technology Co., Ltd. PEG-6000 was used to simulate drought stress (Gonzalez et al., 2024). *M. sativa* L. cv. Gannong No. 3 (G3) was selected as the experimental material in this study. G3 alfalfa is adapted to be grown in irrigated agricultural areas in the interior of Northwest China and the Loess Plateau, and it is a productive variety bred in irrigated areas, with compact and upright plants, many stems and branches, uniform height, medium-sized leaf blades, a dense green leaf color, purple flowers, an early return to green in the spring, and rapid growth in the early stage, but it is poorly resistant to drought.

Seeds of G3 cultivar were supplied by the Grassland Ecosystems Key Laboratory, Ministry of Education of China. Seed planting method and seedling growing environment are the same as our previous method (Wang et al., 2023). The plants were grown in a growth chamber with a day/night temperature of 25/18°C, a light/dark photoperiod of 16/8 h, a photosynthetic light flux density of 450 μmol m^-2^ s^-1^, and a relative humidity of 45%. Alfalfa was grown for 42 days after seeding and then for 7 days in Hoagland solution with or without Spm. In studies of exogenous spermine to enhance plant drought resistance, spermine concentration was mostly controlled between 0.1-1 mM (Hasan et al., 2021). Thus, three concentrations of Spm, 0.1, 0.5 and 1 mM, were applied. Drought treatment initiated on 49th day. All treatments are shown in **Table 1**, each treatment had six pots of alfalfa. When the drought treatment lasted until the 8th day, healthy leaves fully expanded from more than one-half of each branch were collected from plants in all of the above treatment groups, which were then frozen instantly in liquid nitrogen and stored at -80°C for physiological parameter or metabolomic analysis.

**Table 1:** All treatments and specific methods of implementation in the experiment.

Treatments	Explanation
Normal water+ 0 mM Spm	Alfalfa seedlings without Spm treatment were grown in Hoagland nutrient solution.
Normal water+ 0.1 mM Spm	Alfalfa seedlings treated with 0.1 mM Spm were grown in Hoagland nutrient solution.
Normal water+ 0.5 mM Spm	Alfalfa seedlings treated with 0.5 mM Spm were grown in Hoagland nutrient solution.
Normal water+ 1 mM Spm	Alfalfa seedlings treated with 1 mM Spm were grown in Hoagland nutrient solution.
Drought+ 0 mM Spm	Alfalfa seedlings without Spm treatment were grown in a nutrient solution with -1.2 MPa PEG-6000.
Drought+ 0.1 mM Spm	Alfalfa seedlings treated with 0.1 mM Spm were grown in a nutrient solution with -1.2 MPa PEG-6000.
Drought+ 0.5 mM Spm	Alfalfa seedlings treated with 0.5 mM Spm were grown in a nutrient solution with -1.2 MPa PEG-6000.
Drought+ 1 mM Spm	Alfalfa seedlings treated with 1 mM Spm were grown in a nutrient solution with -1.2 MPa PEG-6000.

### 2.2 Measurement and methods

#### 2.2.1 Leaf relative water content

The fresh weight of the leaves was determined immediately after cutting, and then the leaves were placed in beakers with distilled water and left in the dark for 24 h. The saturated weight was measured, and then the dry weight was determined after drying in an oven at a constant temperature of 80°C until a constant weight was reached. The RWC of the leaves was determined by the following equation:


$$\begin{array}{c} \text{RWC=}[\left(\text{fresh weight-dry weight}\right)\\ ÷\left(\text{saturated weight-dry weight}\right)]\times 100\% \end{array}$$

#### 2.2.2 Leaf photosynthetic pigment content

Leaves (0.1 g) were placed in a tube with 5 ml of 95% ethanol for 24 h. Chl extracts were measured for absorbance at 470, 649 and 665 nm for A470, A649 and A665, respectively. The photosynthetic pigment concentration was first calculated according to the following equation: Chla concentration = 13.9 × A665 − 6.88A × A649; Chlb concentration = 24.96 × A649 − 7.32 × A665; Car concentration = (1000 × A470 − 2.05 × Chla concentration − 114.8 × Chlb concentration)/245. The photosynthetic pigment content was then calculated by the following equation: Photosynthetic pigment content = (Photosynthetic pigment concentration × 5 mL) ÷ 0.1g (Arnon, 1949).

#### 2.2.3 Gas exchange parameters

Gas exchange parameters were recorded with the GFS-3000 portable photosynthesis system (Heinz-Walz, Effeltrich, Germany) from 9:00 to 11:00 a.m. in well-lit conditions. Net photosynthetic rate (*P_n_*), transpiration rate (*T_r_*), intracellular CO_2_ concentration (*C_i_*), and stomatal conductance (*g_s_*) were measured using the method of von Caemmerer and Farquhar (1981).

#### 2.2.4 Chl fluorescence parameters

Chl fluorescence was measured using an IMAGING-PAM Chl fluorometer (Heinz-Walz GmbH, Effeltrich, Germany) after 20 min of dark adaptation of the leaves. The *PSII* maximum photochemical efficiency (*Fv/Fm*), PSII photochemical quantum yield (*ФPSII*), electron transfer rate (*ETR*), photochemical burst coefficient (*qP*), and nonphotochemical burst coefficient (*qN*) were calculated based on the method of Li et al. (2018).

#### 2.2.5 Superoxide anion (O_2_^−^·), H_2_O_2_ and MDA contents

The O_2_^−^· and H_2_O_2_ concentrations were analyzed using H_2_O_2_ content assay kits (Suzhou Grace Biotechnology Co., Ltd., SZ, China; http://www.geruisi-bio.com/). The MDA content was determined by the thiobarbituric acid method with reference to Draper and Hadley (1990).

#### 2.2.6 SOD, POD, and CAT activities

The leaf samples (0.1 g) were homogenized in 50 mM phosphate buffer (pH 7.2) containing 4% PVP and 1 mM EDTA. At 4°C, the supernatants were used to test POD, SOD, and CAT activity. The POD, SOD and CAT activities were evaluated using our previous methods (Wang et al., 2023).

#### 2.2.7 AsA-GSH cycle enzyme activities and nonenzymatic antioxidants

The contents of AsA, dehydroascorbic acid (DHA), GSH, and glutathione disulfide (GSSG) as well as the GR and APX activities were determined by commercial assay kits (Suzhou Grace Biotechnology Co., Ltd., SZ, China; http://www.geruisi-bio.com/).

#### 2.2.8 SP, SS, and Pro contents

The SP content was measured by Thomas brilliant blue G-250 staining (Bradford, 1976), the SS content was assayed by anthrone colorimetry (Jan and Roel, 1993), and the free Pro content was determined by acidic ninhydrin colorimetry (Rosen, 1957).

#### 2.2.9 Metabolite extraction and quantification

The fresh leaf samples (100 mg) were separately ground with liquid nitrogen, then the homogenate was resuspended in pre-cooled 80% methanol and vortexed. After incubating for 5 minutes on ice, samples were centrifuged at 15,000 g for 20 minutes at 4°C. Methanol concentration was adjusted to 53% by diluting the supernatant with LC-MS grade water. The samples were transferred to a new Eppendorf tube and centrifuged at 15000 g for 20 minutes at 4°C. The supernatant was then injected into the LC-MS-MS/MS system for analysis.

Novogene Co., Ltd (Beijing, China) conducted UHPLC-MS/MS analyses on a Vanquish UHPLC system (Thermo Fisher, Germany) equipped with an Orbitrap Q Exactive™ HF mass spectrometer or Orbitrap Q Exactive™ HF-X mass spectrometer. The samples were injected on a Hypersil Gold column (100 × 2.1 mm, 1.9 μm) with a linear gradient at a flow rate of 0.2 mL/minute for 12 minutes. Mobile phases A and B for positive and negative polar patterns were 0.1% FA water and methanol, respectively. Solvent gradients were established as follows:1.5 minutes, 2% B; 3 minutes, 2-85% B; 10 minutes, 85-100% B; 10.1 minutes, 100-2% B; and 12 minutes, 2% B. In positive/negative polarity mode, the Q Exactive™ HF mass spectrometer was operated at a spray voltage of 3.5 kV, a capillary temperature of 320°C, a sheath gas flow rate of 35 psi, an aux gas flow rate of 10 L/min, an S-lens RF level of 60 and an aux gas heater temperature of 350°C.

### 2.3 Statistical analysis

Physiological data analysis was performed with GraphPad Prism 8.0.2, and Tukey’s test and two-way ANOVA were used to determine significant differences. *p*<0.05 indicated statistical significance. The principal component analysis (PCA) analysis of physiological data using origin 2021 software. Redundancy analysis (RDA) was performed using Canoco5 software. Fisher discriminant analysis (FDA) was performed using SPSS 25.0 (IBM Corporation, USA) software. Metabolites annotated using Kyoto Encyclopedia of Genes and Genomes (KEGG) database (https://www.genome.jp/kegg/pathway.html), Human Metabolome Database (HMDB) (https://hmdb.ca/metabolites) and LIPIDMaps database (http://www.lipidmaps.org/). The PCA was performed using metaX, a versatile and comprehensive software package for metabolomics analysis. Statistical significance (*p* value) was calculated using univariate analysis (t-test). Metabolites with the variable importance in the projection (VIP) > 1, *p* value< 0.05 and fold change (FC) ≥ 2 or FC ≤ 0.5 were recognized as differentially abundant metabolites (DAMs). Metabolites of interest were filtered using volcano plots based on the log_2_(FC) and -log_10_(*p* value) values of the metabolites via ggplot2 in R. The function of these metabolites and metabolic pathways was investigated using the KEGG database. The metabolic pathways of DAMs were enriched. Metabolic pathways were considered enriched when the ratio satisfied x/n > y/N; metabolic pathways were considered significantly enriched when the p value of the metabolic pathway was< 0.05.

## 3 Results

### 3.1 Plant height and leaf RWC

**Figure 1:**
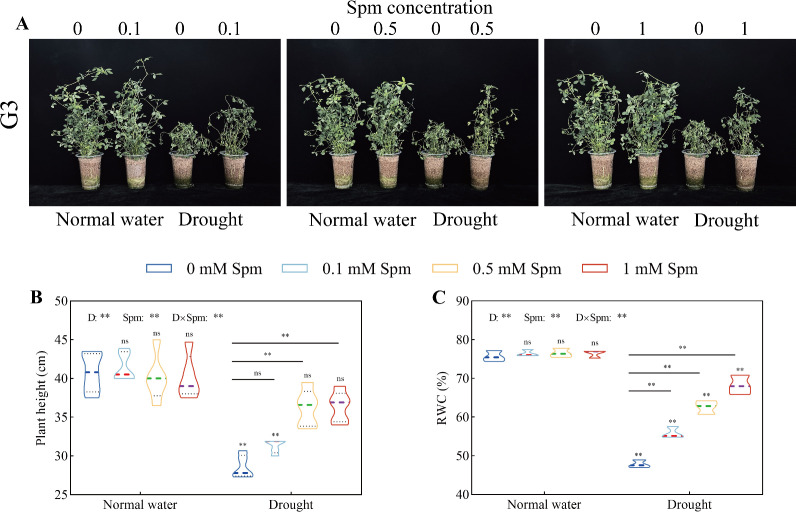
Effects of exogenous Spm application on the height and leaf RWC of G3 alfalfa under normal water conditions and drought stress. **(A)** Pictures of G3 alfalfa growth conditions in all treatments in the experiment. **(B)** Plant height of G3. **(C)** Leaf RWC of G3. “ns”, no significant difference between the normal water conditions without Spm treatment and the other treatments (*p* > 0.05); “*”, significant difference between the normal water conditions without Spm treatment and the other treatments (*p*< 0.05); “**”, extremely significant difference between the normal water treatment without Spm treatment and the other treatments (*p*< 0.01). “ns” no significant difference between the drought conditions without Spm treatment and the drought conditions with Spm treatment (*p* > 0.05); “*”, significant difference between the drought conditions without Spm treatment and the drought conditions with Spm treatment (*p*< 0.05); “**”, extremely significant difference between the drought conditions without Spm treatment and the drought conditions with Spm treatment (*p*< 0.01). Normal water: normal water level treatment group; drought: drought stress treatment group. G3: *Medicago sativa* L. cv. Gannong No. 3.

After drought treatment alone, the plant height and leaf RWC of G3 alfalfa decreased significantly. Spm treatment significantly increased the plant height and leaf RWC of alfalfa plants under drought stress (**Figure 1**). After the 0.5 and 1 mM Spm treatments, the plant height significantly increased by 26.75% and 27.38%, respectively, in G3 alfalfa compared to that under drought stress alone (**Figure 1B**). Compared with those under drought stress alone, the RWC of G3 alfalfa leaves under 0.1, 0.5 and 1 mM Spm treatment significantly increased by 16.73%, 30.85% and 42.61%, respectively (**Figure 1C**).

### 3.2 Leaf photosynthetic pigment contents and ratios

The Chla, Chl(a+b) and Car contents and the Chla/Chlb and Car/Chl(a+b) ratios in the leaves of G3 alfalfa significantly decreased after drought stress alone compared with those under normal water conditions without Spm treatment. Compared with drought treatment alone, the application of Spm under drought stress increased the content of photosynthetic pigments in the alfalfa leaves. The Chla, Chlb, Chl(a+b) and Car contents, Chla/Chlb ratio and Car/Chl(a+b) ratio of G3 alfalfa leaves under drought stress significantly increased by 64.04%, 19.23%, 45.34%, 88.65%, 37.61% and 29.73%, respectively, after the application of 1 mM Spm. This result showed that the application of Spm promoted chl synthesis in leaves and slowed the decomposition and transformation of chl under drought stress, thereby enhancing the photosynthetic ability of alfalfa leaves under drought stress (**Table 2**).

**Table 2:** Effects of exogenous Spm application on the photosynthetic pigment contents and their ratios in G3 alfalfa leaves under normal water conditions and drought stress.

Treatments	Chla	Chlb	Chl(a+b)	Chla/Chlb	Car	Car/Chl(a+ b)
Normal water+0 mM Spm	1.108 ± 0.081	0.517 ± 0.022	1.625 ± 0.062	2.149 ± 0.147	0.163 ± 0.010	0.100 ± 0.003
Normal water+0.1 mM Spm	1.092 ± 0.048 ns	0.540 ± 0.022ns	1.632 ± 0.054ns	2.024 ± 0.119ns	0.164 ± 0.009ns	0.100 ± 0.006ns
Normal water+0.5 mM Spm	1.105 ± 0.047ns	0.517 ± 0.033ns	1.622 ± 0.079ns	2.141 ± 0.060ns	0.161 ± 0.006ns	0.099 ± 0.001ns
Normal water+1 mM Spm	1.121 ± 0.069ns	0.523 ± 0.017ns	1.645 ± 0.066ns	2.144 ± 0.168ns	0.167 ± 0.010ns	0.101 ± 0.003ns
Drought+0 mM Spm	0.616 ± 0.020**	0.441 ± 0.015*	1.057 ± 0.035**	1.396 ± 0.015**	0.079 ± 0.003**	0.075 ± 0.005**
Drought+0.1 mM Spm	0.822 ± 0.033** **	0.487 ± 0.020ns ns	1.308 ± 0.052** **	1.688 ± 0.033** ns	0.107 ± 0.005** **	0.082 ± 0.006** ns
Drought+0.5 mM Spm	0.886 ± 0.040** **	0.489 ± 0.023ns ns	1.375 ± 0.045** **	1.815 ± 0.116* **	0.128 ± 0.006** **	0.093 ± 0.006ns **
Drought+1 mM Spm	1.010 ± 0.059ns **	0.526 ± 0.016ns **	1.536 ± 0.065ns **	1.921 ± 0.114ns **	0.150 ± 0.006ns **	0.098 ± 0.001ns **
D	**	**	**	**	**	**
Spm	**	*	**	**	**	**
D×Spm	**	*	**	**	**	**

“ns”, no significant difference between the normal water conditions without Spm treatment and the other treatments (*p* > 0.05); “*”, significant difference between the normal water conditions without Spm treatment and the other treatments (*p*< 0.05); “**”, extremely significant difference between the normal water treatment without Spm treatment and the other treatments (*p*< 0.01). “ns”, no significant difference between the drought conditions without Spm treatment and the drought conditions with Spm treatment (*p* > 0.05); “*”, significant difference between the drought conditions without Spm treatment and the drought conditions with Spm treatment *p*< 0.05); “**”, extremely significant difference between the drought conditions without Spm treatment and the drought conditions with Spm treatment (*p*< 0.01). Normal water: normal water treatment group; drought: drought stress treatment group. G3: *Medicago sativa* L. cv. Gannong No. 3.

### 3.3 Gas exchange parameters

**Figure 2:**
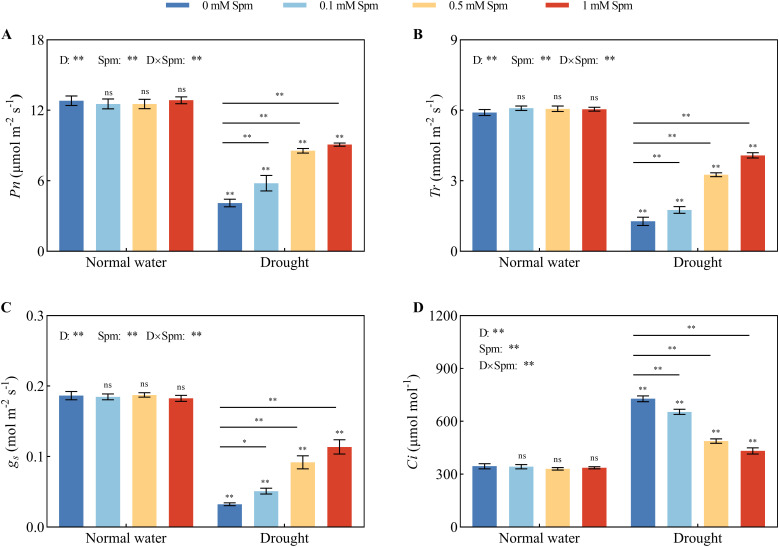
Effects of exogenous Spm application on the gas exchange parameters of G3 alfalfa under normal water conditions and drought stress. **(A)** *P_n_* of G3. **(B)** *T_r_* of G3. **(C)** *g_s_* of G3. **(D)** *C_i_* of G3. Significance markers are explained in the caption of **Figure 1**.

After drought stress alone, the *P_n_*, *T_r_*, and *g_s_* values of the leaves significantly decreased compared with those grown under normal water conditions without Spm treatment. The addition of Spm increased the *P_n_*, *T_r_*, and *g_s_* values of alfalfa plants under drought stress (**Figures 2A–C**). The *C_i_* values of alfalfa leaves under drought stress alone were significantly greater than those under normal water conditions without Spm treatment. The addition of Spm significantly reduced the *C_i_* of alfalfa leaves under drought stress. Compared with drought treatment alone, after 0.1, 0.5, and 1 mM Spm treatment, *C_i_* decreased by 10.18%, 32.95%, and 40.63%, respectively, in G3 alfalfa leaves (**Figure 2D**). These results indicated that drought stress limited photosynthesis in alfalfa, while Spm application improved the values of gas exchange parameters in alfalfa leaves under drought stress. Specifically, exogenous Spm application increased the *P_n_*, *g_s_* and *T_r_* of leaves and decreased the *C_i_* of leaves.

### 3.4 Chl fluorescence parameters

**Figure 3:**
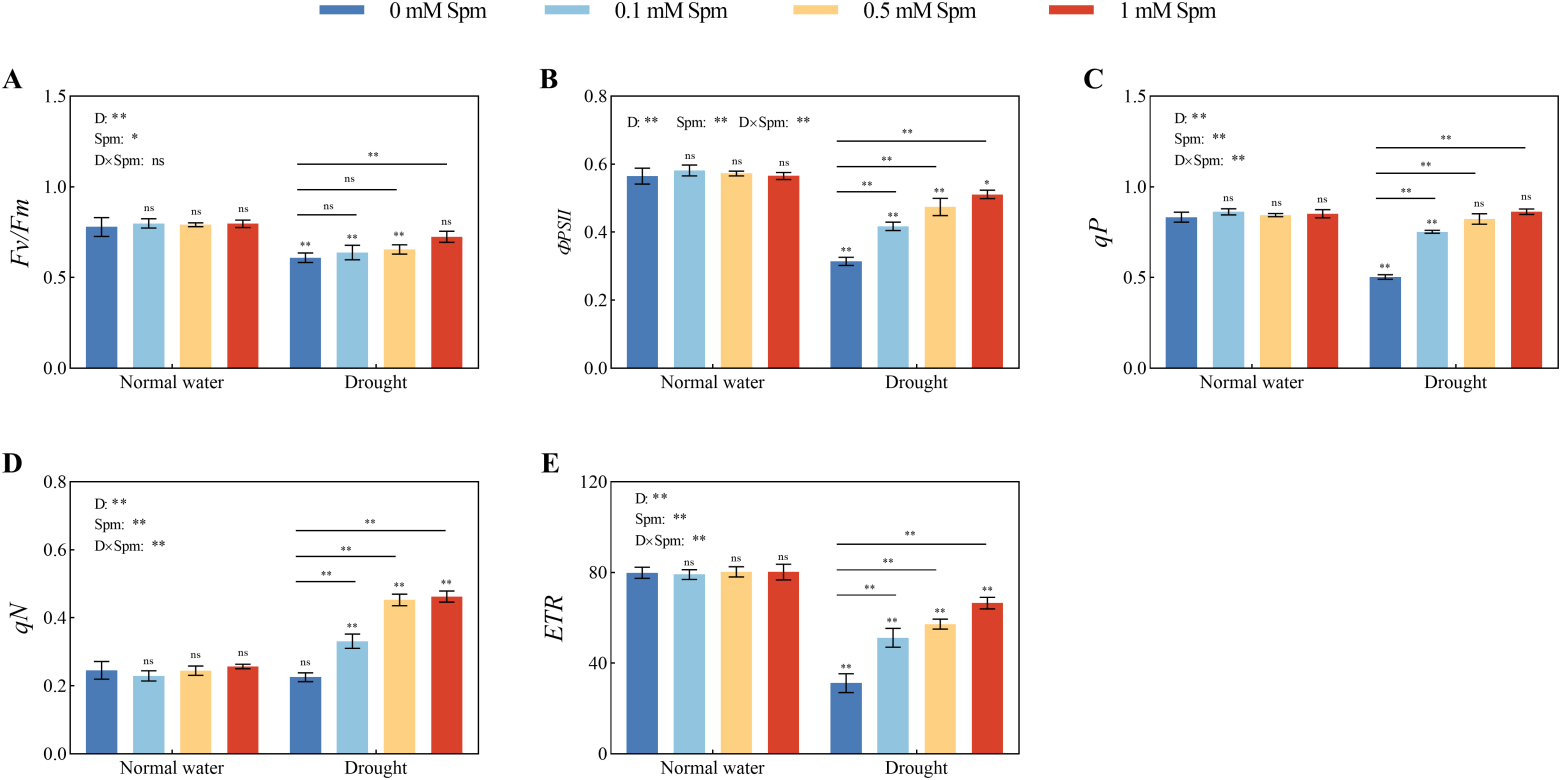
Effects of exogenous Spm application on the Chl fluorescence parameters of G3 alfalfa under normal water conditions and drought stress. **(A)** *Fv/Fm* of G3. **(B)** *ФPSII* of G3. **(C)** *qP* of G3. **(D)** *qN* of G3. **(E)** *ETR* of G3. Significance markers are explained in the caption of **Figure 1**.

As shown in **Figure 3**, after drought treatment alone, the *Fv/Fm*, *ΦPSII*, *qP* and *ETR* values decreased and were significantly lower than those under normal water conditions without Spm treatment, but the *qN* value did not change significantly. Drought stress decreased in the chl fluorescence parameters of alfalfa leaves, which is extremely unfavorable for light energy absorption and utilization by leaves. Compared with those under normal water conditions without Spm treatment, the *Fv/Fm*, *ФPSII*, *qP* and *ETR* in G3 alfalfa after drought treatment alone decreased by 21.75%, 44.34%, 39.61% and 60.94%, respectively. After the application of Spm under drought stress, the *Fv/Fm*, *ФPSII*, *qP*, *ETR* and *qN* values of the alfalfa leaves increased. The optimal Spm concentration for improving the chl fluorescence parameters of G3 alfalfa leaves was 1 mM. The *Fv/Fm*, *ФPSII*, *qP*, *ETR* and *qN* values of G3 alfalfa leaves under drought stress significantly increased by 18.84%, 62.54%, 71.60%, 113.24% and 105.10%, respectively, after the application of 1 mM Spm in G3 alfalfa. The above results showed that alfalfa could maintain high PSII photochemical efficiency under drought stress with the application of Spm.

### 3.5 O_2_^−^·, H_2_O_2_ and MDA contents

**Figure 4:**
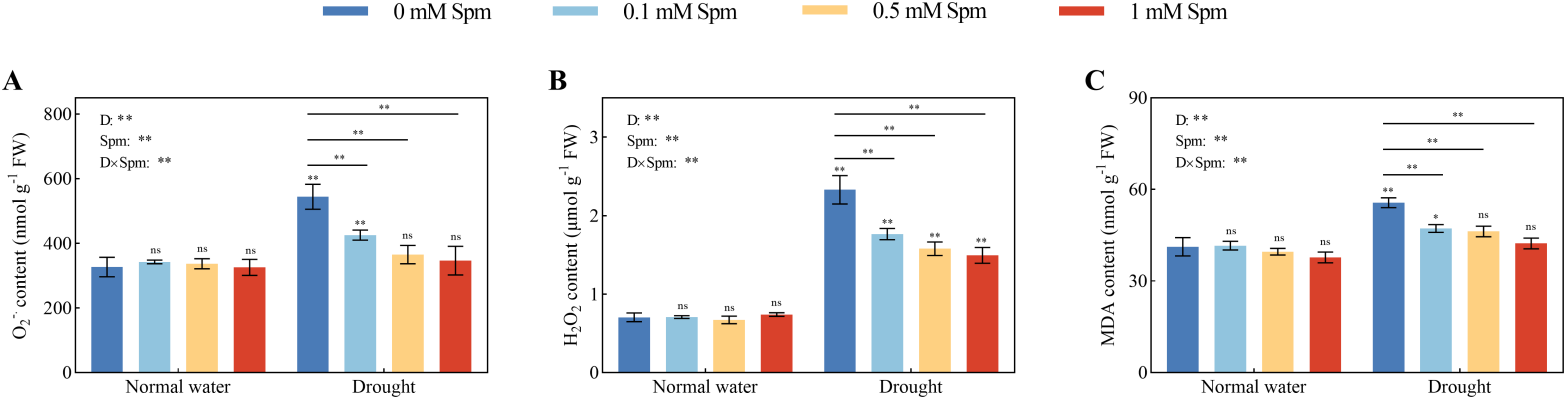
Effects of exogenous Spm application on the O_2_^−^·, H_2_O_2_ and MDA contents of G3 alfalfa leaves under normal water conditions and drought stress. **(A)** O_2_^−^· content of G3. **(B)** H_2_O_2_ content of G3. **(C)** MDA content of G3. Significance markers are explained in the caption of **Figure 1**.

The O_2_^−^· and H_2_O_2_ contents in G3 alfalfa increased by 66.53% and 230.52%, respectively, after drought treatment alone compared to those in plants grown under normal water conditions without Spm treatment. The application of Spm had no effect on the O_2_^−^·or H_2_O_2_ content of alfalfa leaves under normal water conditions. However, exogenous Spm application can reduce the contents of O_2_^−^·and H_2_O_2_ in leaves under drought stress. Compared with drought treatment alone, all concentrations of Spm (0.1, 0.5 and 1 mM) significantly decreased the O_2_^−^· and H_2_O_2_ contents in G3. Among the treatments, 1 mM Spm application had the greatest effect, and the O_2_^−^· and H_2_O_2_ contents in G3 alfalfa leaves treated with 1 mM Spm under drought stress decreased by 36.31% and 35.82%, respectively (**Figures 4A, B**). Drought stress caused the leaf MDA content to increase in alfalfa plants. Compared with that in plants grown under normal water conditions without Spm treatment, the MDA content in the leaves of G3 alfalfa under drought alone increased by 34.87%. Under normal water conditions, the application of three concentrations of Spm had no effect on the MDA content in alfalfa leaves. The application of Spm significantly reduced the MDA content of alfalfa plants under drought stress. The MDA content of G3 alfalfa leaves was significantly reduced by 15.16%, 16.87% and 23.93% after the application of 0.1, 0.5 and 1 mM Spm, respectively, under drought stress (**Figure 4C**).

### 3.6 SOD, POD and CAT activities

**Figure 5:**
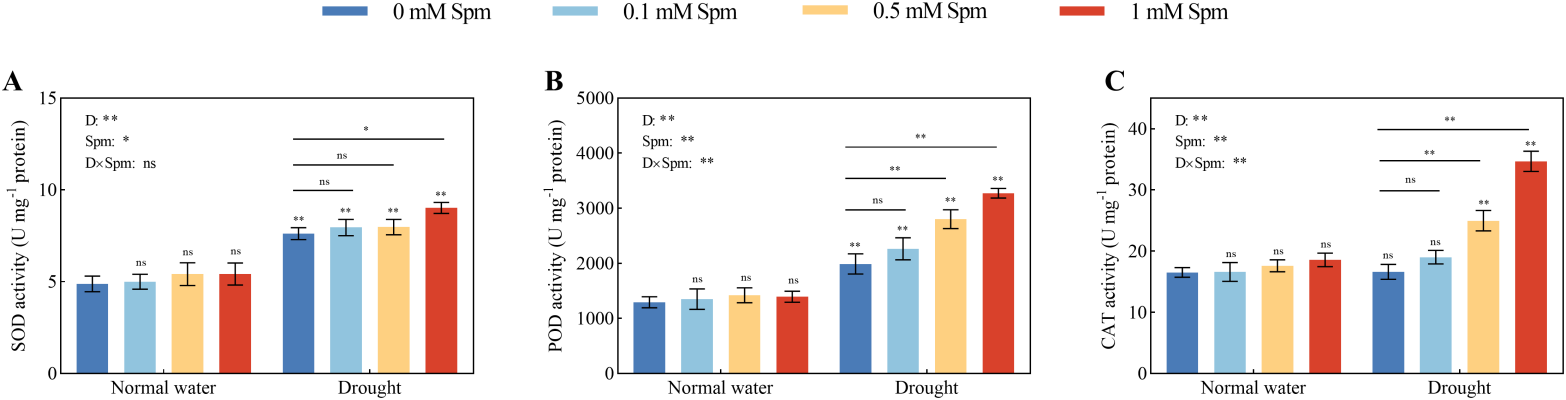
Effects of exogenous Spm application on SOD, POD and CAT activities in G3 alfalfa leaves under normal water conditions and drought stress. **(A)** SOD activity in G3. **(B)** POD activity in G3. **(C)** CAT activity in G3. Significance markers are explained in the caption of **Figure 1**.

Compared with those in plants grown under normal water conditions without Spm treatment, the SOD and POD activities in the leaves of G3 alfalfa exposed to drought stress alone increased by 55.89% and 53.53%, respectively, while there was no significant difference in the leaf CAT activity. Under normal water conditions, exogenous Spm application had no effect on the SOD, POD or CAT enzyme activity in alfalfa leaves. The activities of antioxidant enzymes increased in response to exogenous Spm treatment under drought stress. Compared with drought treatment alone, the addition of 1 mM Spm under drought stress significantly increased the SOD, POD and CAT activities of G3 alfalfa leaves by 18.46%, 64.64% and 108.42%, respectively (**Figure 5**). The above results indicated that exogenous Spm application could increase the SOD, POD and CAT activities of alfalfa under drought stress, and the increase in POD and CAT activities was more notable than that of SOD.

### 3.7 AsA-GSH cycle enzyme activities and nonenzymatic antioxidant levels

**Figure 6:**
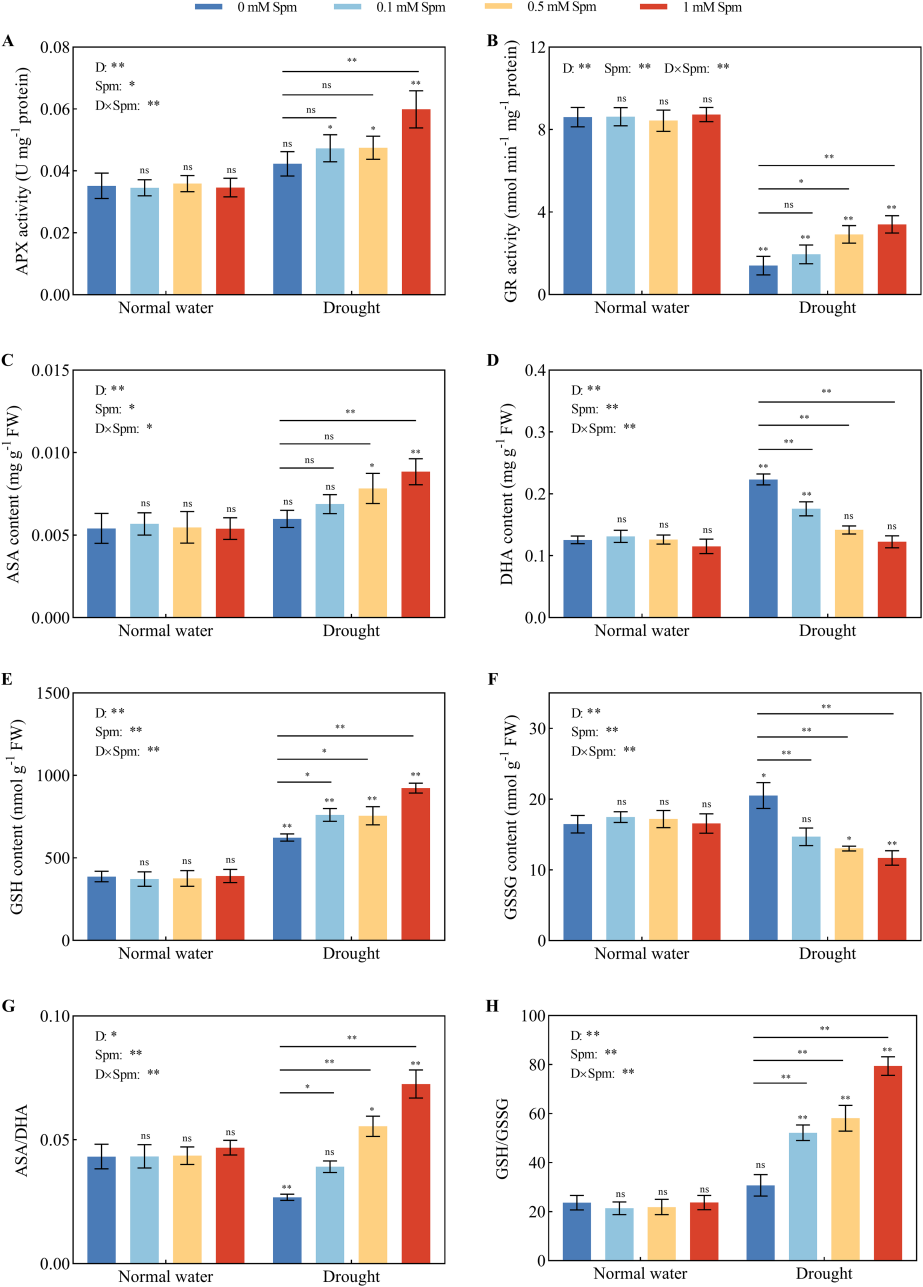
Effects of exogenous Spm application on AsA-GSH cycle enzyme activities and nonenzymatic antioxidants in G3 alfalfa leaves under normal water conditions and drought stress. **(A)** APX activity of G3. **(B)** GR enzyme activity of G3. **(C)** ASA content of G3. **(D)** DHA content of G3. **(E)** GSH content of G3. **(F)** GSSG content of G3. **(G)** ASA/DHA of G3. **(H)** GSH/GSSG ratio of G3. Significance markers are explained in the caption of **Figure 1**.

Compared with that under normal water conditions without Spm treatment, there was no significant difference in the APX activity of G3 alfalfa leaves under drought stress alone; the GR activity of G3 alfalfa leaves was significantly reduced by 83.68% under drought stress alone. The APX activity of G3 alfalfa leaves under drought stress significantly increased by 41.62% after the application of 1 mM Spm. Compared with drought stress alone, the addition of 0.1 mM Spm under drought stress had no significant effect on the GR enzyme activity in alfalfa, while the GR enzyme activity in G3 alfalfa leaves significantly increased by 107.83% and 142.21% after the application of 0.5 and 1 mM Spm, respectively (**Figures 6A, B**). Compared with normal water conditions without Spm treatment, drought stress alone significantly increased the DHA content of G3 alfalfa leaves by 77.75% but had no significant effect on the AsA content, resulting in a 38.07% reduction in the ASA/DHA ratio. Under drought stress, exogenous Spm application increased the AsA content and decreased the DHA content in the leaves of alfalfa plants, which in turn led to an increase in the AsA/DHA ratio. Under drought stress, the application of 1 mM Spm significantly increased the AsA content and AsA/DHA ratio of the G3 alfalfa leaves by 47.74% and 170.84%, respectively (**Figures 6C, D, G**). Compared with normal water conditions without Spm treatment, drought stress alone significantly increased the GSH and GSSG contents of G3 alfalfa leaves by 60.89% and 24.58%, respectively. The GSH/GSSG ratio of G3 alfalfa leaves was not significantly different from that of plants grown under normal water conditions without Spm treatment. The addition of Spm under drought stress increased the GSH content and decreased the GSSG content in alfalfa leaves, resulting in a marked increase in the GSH/GSSG ratio. The GSH/GSSG ratio of the G3 alfalfa leaves significantly increased by 70.07%, 89.22% and 158.68%, respectively, in response to the application of 0.1, 0.5 and 1 mM Spm under drought stress (**Figures 6E, F, H**). The above results indicated that the addition of exogenous Spm under drought stress increased the activities of antioxidant enzymes and the contents of nonenzymatic antioxidants in leaves, which enhanced the ability of leaves to scavenge the large amounts of O_2_^−^· and H_2_O_2_ produced by drought stress, thus effectively alleviating the oxidative damage caused by drought stress on leaves. These results indicated that exogenous Spm enhanced SOD, POD and CAT activities and the ASA-GSH cycle to strengthen the antioxidant system of alfalfa under drought stress, thus reducing the production of ROS.

### 3.8 Osmoregulatory compounds content

**Figure 7:**
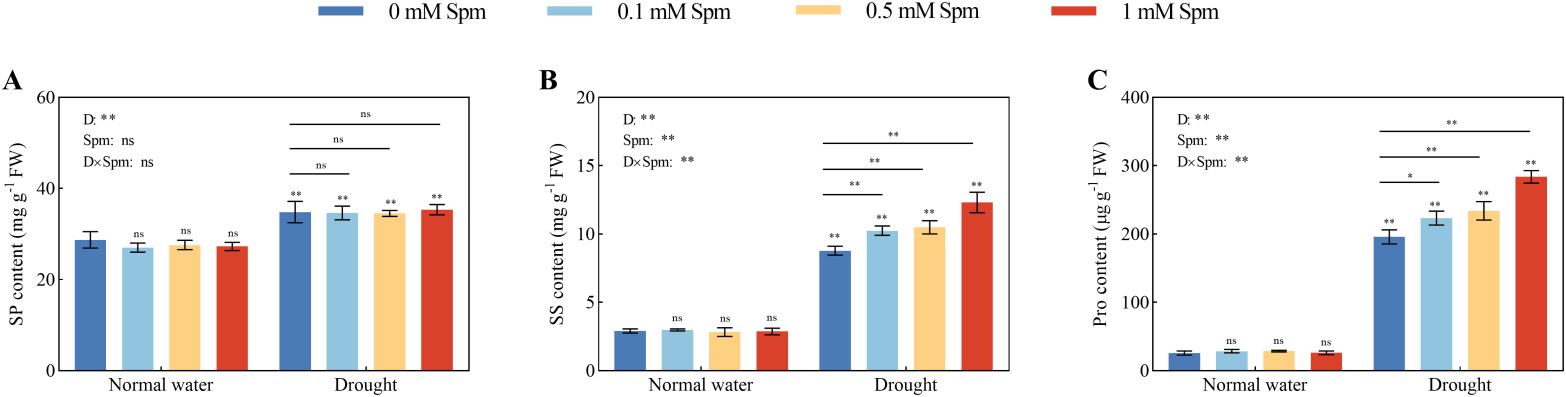
Effects of exogenous Spm application on the SP, SS and Pro contents in G3 alfalfa leaves under normal water conditions and drought stress. **(A)** SP content of G3. **(B)** SS content of G3. **(C)** Pro content of G3. Significance markers are explained in the caption of **Figure 1**.

Drought stress alone induced significant increases in the Pro, SP and SS contents in alfalfa leaves compared to those in plants grown under normal water conditions without Spm treatment. The application of Spm had no effect on the levels of Pro, SP or SS in alfalfa leaves under normal water conditions; moreover, Spm application under drought stress had no effect on the SP content in alfalfa leaves (**Figure 7A**). The application of 0.1, 0.5 and 1 mM Spm significantly altered the Pro and SS contents of the G3 alfalfa under drought stress, with 1 mM Spm being the most effective, which significantly increased the Pro and SS contents of the G3 alfalfa by 44.90% and 40.15%, respectively (**Figures 7B, C**). The results showed that exogenous Spm application increased the contents of osmoregulatory substances in alfalfa exposed to drought stress, thereby decreasing the osmotic potential and increasing the water retention of the cells, which in turn enhanced its drought resistance.

### 3.9 Comprehensive analysis of the effects of exogenous Spm application on the physiological parameters of G3 alfalfa leaves under drought stress

**Figure 8:**
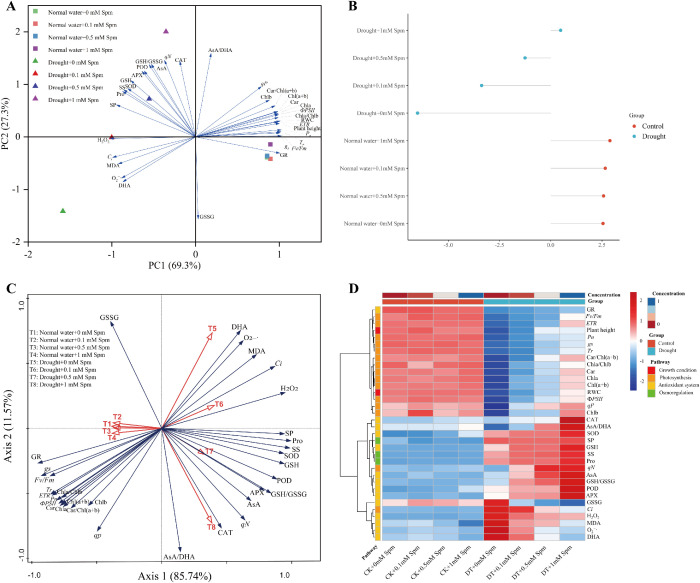
Comprehensive analysis of the effects of exogenous Spm application on the physiological parameters of G3 alfalfa leaves under drought stress. **(A)** PCA analysis of physiological indicators under all treatments. **(B)** PCA scores for all treatments. **(C)** RDA analysis of all treatments and physiological indicators. **(D)** Physiological indicators heatmap.

We performed PCA, RDA and cluster heatmap analysis of the above physiological parameters. PCA showed that principal component 1 (PC1) and PC2 accounted for 96.6% of the total data variance (**Figure 8A**). H_2_O_2_ content, *g_s_*, *P_n_*, *T_r_*, RWC, *Fv/Fm*, GR activity, plant height, *ФPSII*, *C_i_* and Chla/b ratio (69.3%) were the major contributors to PC1, and most of the indices were related to photosynthesis in alfalfa. PC2 (27.3%) was significantly influenced by ASA/DHA ratio, GSSG activity, *qN*, CAT activity, ASA content and GSH/GSSG ratio, and most of the indicators were related to the antioxidant system of alfalfa. PCA showed that the main difference between the normal water and drought stress treatments was in PC1, where the normal water and stress treatment groups were clearly separated. Under normal water conditions, the Spm treatment groups were clustered with their respective untreated groups, suggesting that the application of Spm under normal water conditions had no effect on alfalfa. In contrast, there was differential performance on PC2 between drought stress alone and Spm application under drought stress, and the individual Spm treatments of G3 alfalfa ranked 1 mM>0.5 mM>0.1 mM>0 mM on PC2. Moreover, we calculated the PCA scores for each treatment. The results of the composite score were consistent with the above results (**Figure 8B**).

To investigate the correlation between the different treatments and each physiological index, we performed RDA. RDA showed that the percentage of cumulative variance explained by the relationships between the different treatments and the physiological indices was 97.31%, indicating that the results are highly representative (**Figure 8C**). The cosine of the angle between each indicator and treatment in the RDA chart indicates the correlation between them. The smaller the angle is, the greater the correlation; if the arrows are in the same direction, there is a positive correlation; if they are reversed, there is a negative correlation; if the angle is close to the right, there is little correlation. The RDA results showed that drought stress treatment alone (T5) was negatively correlated with the photosynthetic index and had a strong positive correlation with O_2_^−^· and MDA contents, which indicated that drought stress severely limited photosynthesis and produced a large amount of ROS, with severe damage to the cell membranes of alfalfa. Drought+1 mM Spm treatment (T8) was positively correlated with CAT activity, ASA content, ASA/DHA ratio, APX activity and GSH/GSSG ratio and negatively correlated with GSSG content. Similar results were demonstrated by our clustered heatmap.

Based on the angle between treatments and indicators and the length of the projection of indicators on treatments in the RDA plot of **Figure 8C**, we extracted the physiological indicator groups and key indicators affecting the addition of Spm treatment under drought stress, as shown in **Table 3**. Under drought stress alone, the oxidative indicators such as DHA content, O_2_^−^· content, MDA content, *C_i_*, H_2_O_2_ content and GSSG content were high, and photosynthetic indicators were severely inhibited. After the addition of 0.1 mM Spm under drought stress, the MDA content, *C_i_*, H_2_O_2_ content and GSSG content decreased, and the contents of osmotic regulators such as SP, Pro, and SS gradually increased compared with those under drought stress alone, but the photosynthetic indices were still severely inhibited. The addition of 0.5 mM Spm under drought stress increased the activities of SOD, POD, and CAT as well as the ASA-GSH cycle. After the addition of 1 mM Spm under drought stress, SOD, POD, and CAT activities and the ASA-GSH cycle were further enhanced.

**Table 3:** Physiological indicator groups and key indicators affected by Spm treatment under drought stress summarized based on RDA analysis.

Indicators	Correlation	Drought+0 mM Spm	Drought+0.1 mM Spm	Drought+0.5 mM Spm	Drought+1mM Spm
Indicator groups	Positive	DHA, O_2_^−^·, MDA, *C_i_*, H_2_O_2_, GSSG	MDA, *C_i_*, H_2_O_2_, GSSG. SP, Pro, SS	SOD, GSH, POD, GSH/GSSG, APX, ASA, *qN*, CAT	GSH/GSSG, APX, ASA, CAT, ASA/DHA, *qN*
	Negative	GR, g_s_, *Fv/Fm*, *T_r_*, *ETR*, Chla, Chlb, Chl(a+b), Chla/Chlb, Car, Car/Chl(a+b), *ФPSII* *P_n_*, *qp*, ASA/DHA, CAT, *qN*	GR, *g_s_*, *Fv/Fm*, *T_r_*, *ETR*, Chla, Chlb, Chl(a+b), Chla/Chlb, Car, Car/Chl(a+b), *ФPSII* *P_n_*, *qp*, ASA/DHA	GSSG, GR	GSSG, GR, DHA, O_2_^−^·
Key indicators	Positive	DHA, O_2_^−^·	*C_i_*, H_2_O_2_	GSH/GSSG, APX	CAT
	Negative	*qp*	*g_s_*, *Fv/Fm*	GSSG	GSSG

As shown in **Table 4**, we used FDA to obtain key physiological indicators that distinguish between drought stress alone and drought stress plus 1 mM Spm treatment in G3 alfalfa leaves. A discriminant function was extracted to explain the difference in the G3 alfalfa response to drought stress treatment and drought stress with Spm treatment. The variables included in the discriminant function were *qp*, H_2_O_2_ content, CAT activity and DHA content.

**Table 4:** Results of FDA of G3 alfalfa under drought stress treatment and drought stress treatment + 1 mM Spm treatment.

Standardized typical discriminant function coefficients	
*qp*	27.556
H_2_O_2_	-10.641
CAT	28.189
DHA	-2.737

### 3.10 Metabolomic analysis

#### 3.10.1 Screening for DAMs

**Figure 9:**
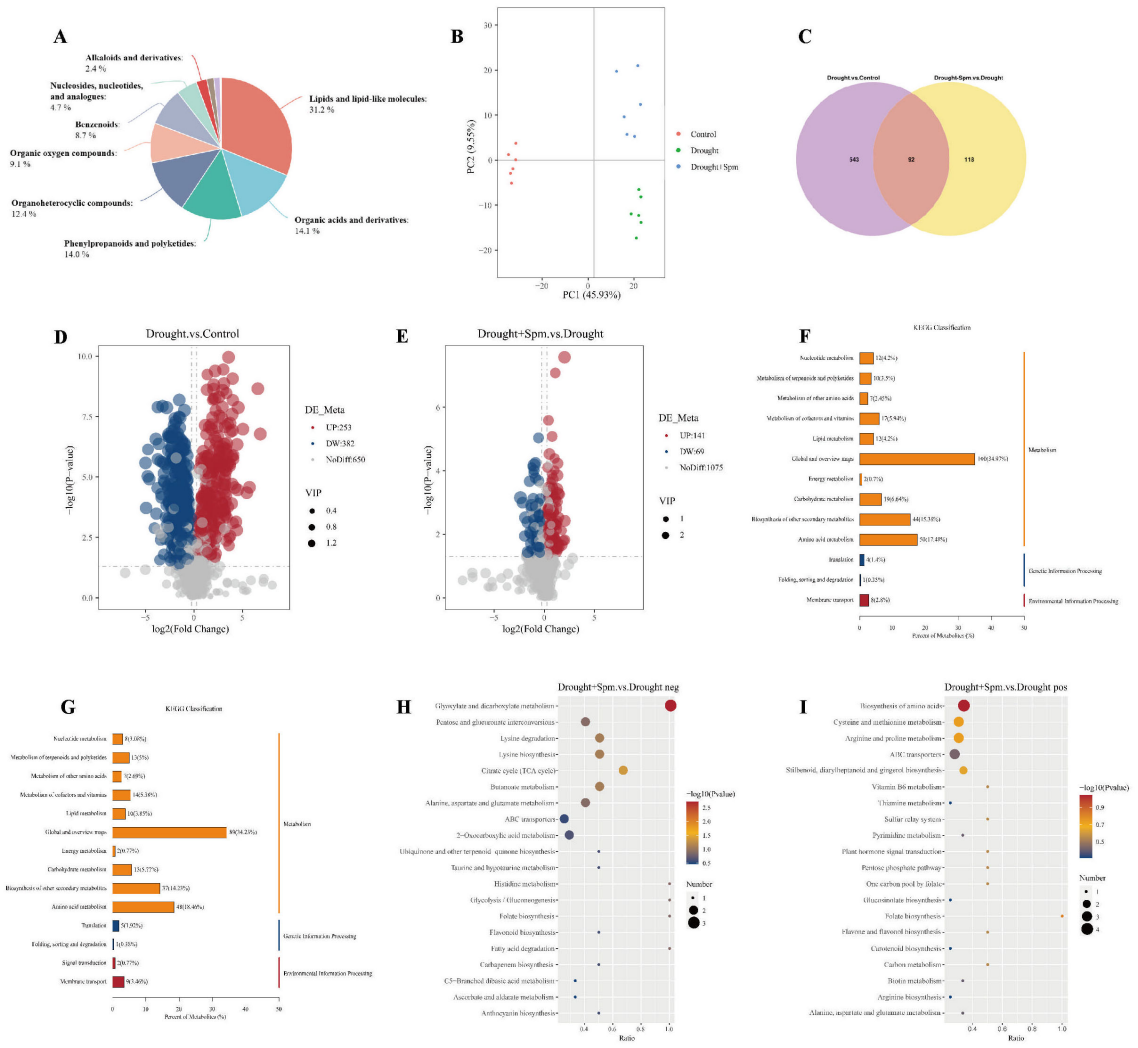
**(A)** Metabolite classification pie chart. **(B)** PCA of total samples. **(C)** Venn diagram analysis of DAMs. **(D)** DAMs volcano plots for drought vs. normal water. **(E)** DAMs volcano plots for drought+Spm vs. normal water. **(F)** KEGG classification analysis for drought vs. normal water. **(G)** KEGG classification analysis for drought+Spm vs. normal water. **(H)** KEGG enrichment bubble plots of drought+Spm vs. normal water in negative ionization mode. **(I)** KEGG enrichment bubble plots of drought+Spm vs. normal water in positive ionization mode.

**Figure 10:**
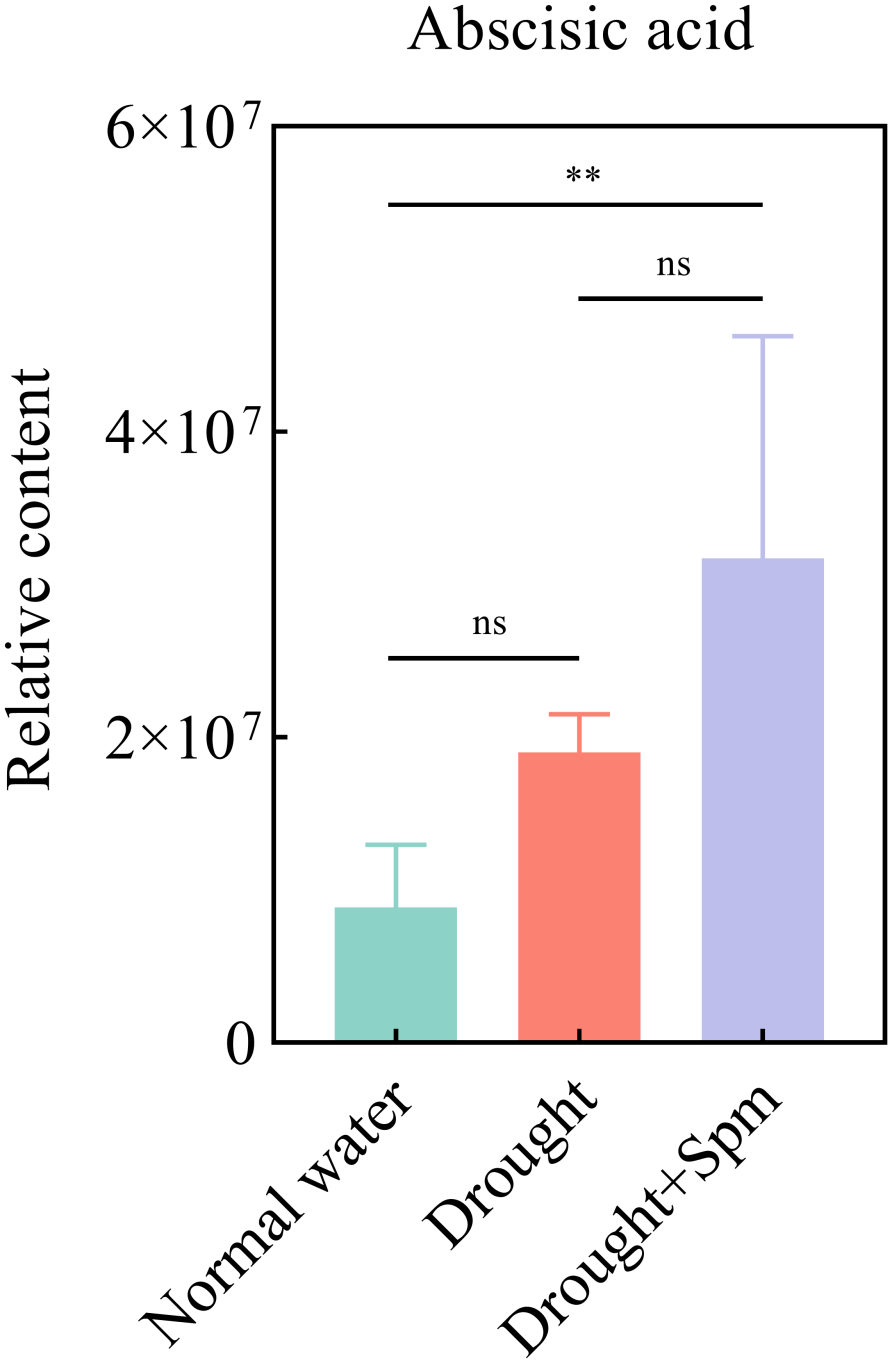
Effect of exogenous Spm application on the ABA content of G3 alfalfa leaves under drought stress. “ns”, no significant difference between the two treatments (*p* > 0.05); “*”, significant difference between the two treatments (*p*< 0.05); “**”, extremely significant difference between the two treatments (*p*< 0.01).

To further explore the mechanism by which exogenous Spm application improves drought tolerance in alfalfa, we performed nontargeted metabolomic analyses on three treatments, namely, normal water, drought and drought+1 mM Spm, in the G3 cultivar. A total of 991 metabolites were detected based on the LC−MS/MS nontargeted metabolomics assay. These metabolites were mainly divided into lipids and lipid−like molecules (31.2%), organic acids and derivatives (14.1%), phenylpropanoids and polyketides (14.0%), organoheterocyclic compounds (12.4%), organic oxygen compounds (9.1%), benzenoids (8.7%), nucleosides, nucleotides and analogs (4.7%), and alkaloids and derivatives (2.4%) (**Figure 9A**). We quantified the relative levels of metabolites measured in all treatments and performed PCA (**Figure 9B**). The PCA results indicated different separation trends between the treatment groups, suggesting that there were differences between the metabolomes of the treatments. As shown in **Figure 9B**, the first two PCs explained 55.48% of the total variation. PC1 accounted for 45.93% of the total data variance, while PC2 explained 9.55% of the data variance for the entire dataset. To screen for DAMs in the drought *vs*. normal water and drought+Spm *vs*. drought comparison groups, we screened for DAMs with a VIP > 1.0, an FC > 1.2 or< 0.833 and a p value< 0.05. As shown in **Figures 9C–E**, a total of 635 DAMs were identified in the drought *vs*. normal water conditions comparison, of which 253 substances were upregulated and 382 substances were downregulated; 210 DAMs were identified in the drought+Spm *vs*. drought comparison, of which 141 substances were upregulated and 69 substances were downregulated. The KEGG classification results showed that abscisic acid (ABA) was involved in signal transduction in the drought+Spm *vs*. drought and the drought *vs*. normal water comparisons (**Figures 9F, G**). Compared with the normal water treatment, drought stress did not significantly affect the ABA content of alfalfa leaves, but the ABA content significantly increased in response to exogenous Spm treatment (**Figure 10**). Differential metabolic pathways were identified through KEGG analysis, and the top twenty enriched KEGG pathways in the drought+Spm treatment group compared with the drought treatment group are shown in **Figures 9H, I**. We focused on two pathways associated with drought tolerance in plants, the TCA cycle and arginine and pro metabolism.

#### 3.10.2 The relative contents of substances related to the TCA cycle and arginine and Pro metabolism

**Figure 11:**
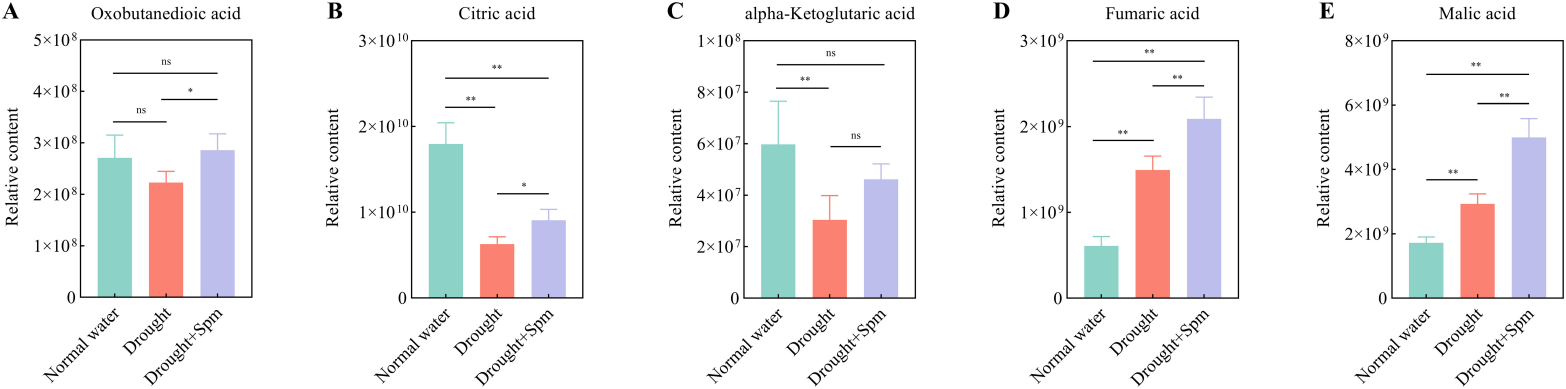
Effect of exogenous Spm application on TCA cycle intermediates in G3 alfalfa leaves under drought stress. **(A)** Oxobutanedioic acid content. **(B)** Citric acid content. **(C)** Alpha-ketoglutaric acid content. **(D)** Fumaric acid content. **(E)** Malic acid content. Significance markers are explained in the caption of **Figure 10**.

**Figure 12:**
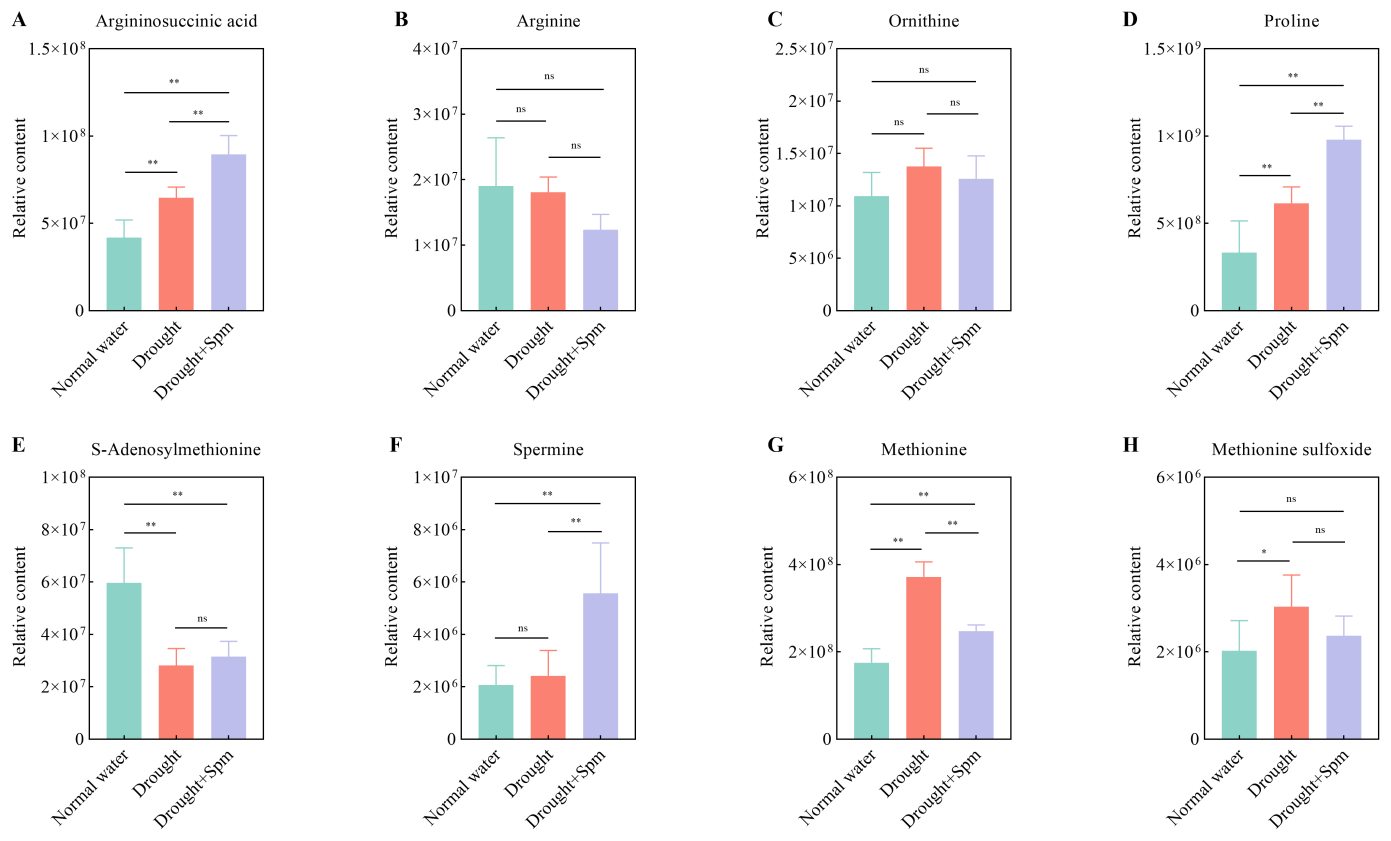
Effect of exogenous Spm application on arginine and proline metabolism intermediates in G3 alfalfa leaves under drought stress. **(A)** Argininosuccinic acid content. **(B)** Arginine content. **(C)** Ornithine content. **(D)** Proline content. **(E)** S-adenosylmethionine content. **(F)** Spm content. **(G)** Methionine content. **(H)** Methionine sulfoxide content. Significance markers are explained in the caption of **Figure 10**.

The TCA cycle mainly includes oxobutanedioic acid, citric acid, alpha-ketoglutaric acid, fumaric acid and malic acid. Drought stress significantly reduced the contents of citric acid and alpha-ketoglutaric acid in alfalfa leaves, while the application of Spm under drought stress significantly increased the contents of oxobutanedioic acid, citric acid, fumaric acid and malic acid in alfalfa leaves (**Figure 11**). With respect to arginine and Pro metabolism, the addition of Spm under drought stress significantly increased the argininosuccinic acid, Pro and Spm contents compared to those under drought stress alone (**Figures 12A–F**). Compared with the normal water treatment, drought stress significantly increased the contents of methionine (Met) and methionine sulfoxide (MetSO), but Spm application decreased the accumulation of Met and MetSO (**Figures 12G, H**).

#### 3.10.3 Combined analysis of the TCA cycle and relative contents of arginine and Pro metabolic pathway-related substances and ABA

**Figure 13:**
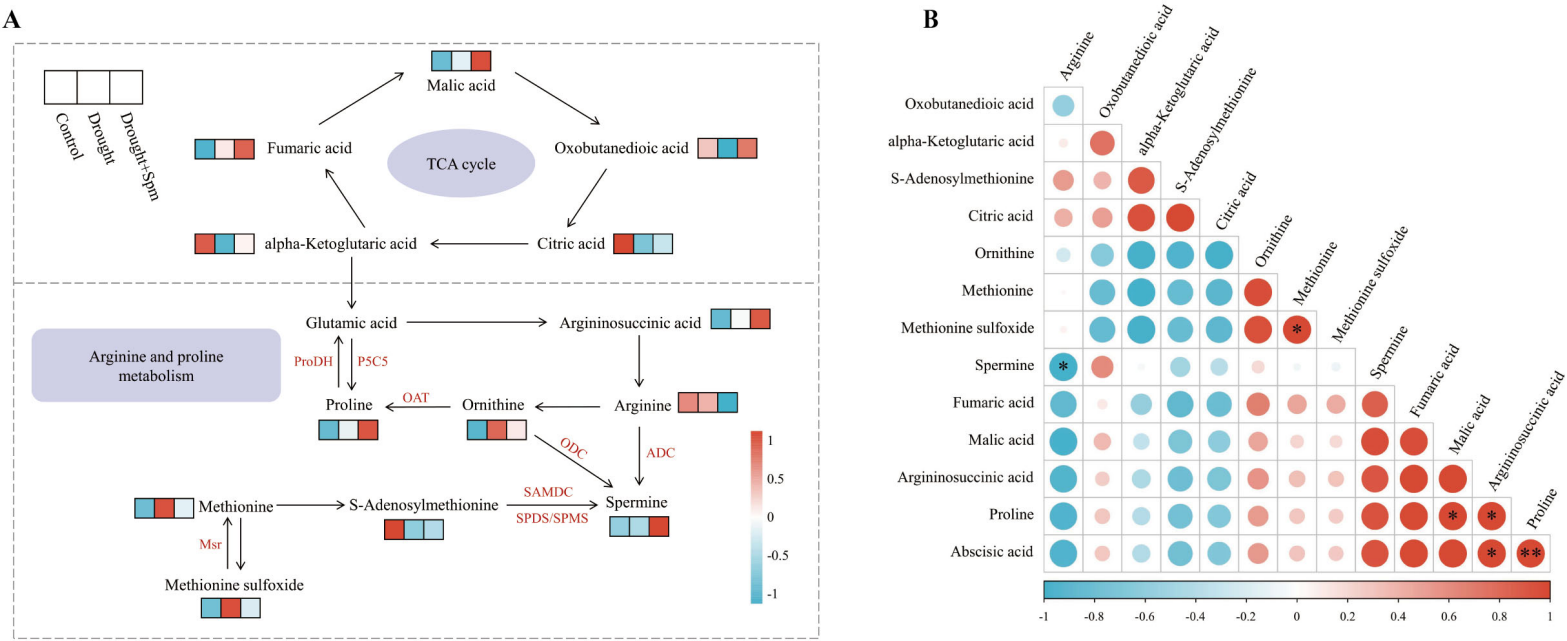
**(A)** Heatmap of the TCA cycle and arginine and proline metabolism. **(B)** Correlation analysis of TCA cycle intermediates, arginine and proline metabolism intermediates and ABA. Correlations are significant at *p< 0.05 and **p< 0.01.

To more clearly show the changes in the TCA cycle and arginine and Pro metabolism in alfalfa after Spm application under drought stress, we generated a heatmap of the relative contents of related substances involved in the TCA cycle and arginine and Pro metabolism (**Figure 13A**). In addition, we performed correlation analysis on the relative contents of substances related to ABA, the TCA cycle, and arginine and Pro metabolism, and the results showed that arginine content was significantly negatively correlated with Spm content (*p*< 0.05) and that the ABA content was significantly positively correlated with the Pro content (*p*< 0.01) (**Figure 13B**).

## 4 Discussion

Globally, drought is a problematic stressor that can disproportionately reduce crop production. PEG-6000 is a polymer that is often used as a water stress agent in drought simulations. It is highly hydrophilic and able to seize water, affecting the function of the plant’s transport tissues and thus causing drought stress to plants (Zhang and Shi, 2018). Therefore, in this study, we used PEG-6000 to simulate drought stress.

### 4.1 Spm increased plant height and leaves RWC

In the present study, drought stress decreased the plant height and RWC of leaves. However, exogenous Spm application increased plant height of alfalfa under drought stress. This may be attributed to the effective regulation of nitrogen metabolism by PAs (Zhang et al., 2013b). In addition, exogenous PA slowed down the decrease of RWC in alfalfa leaves subjected to drought stress. A previous study showed that exogenous PAs increased RWC in lettuce under drought stress (Liu et al., 2018). This is consistent with our results.

### 4.2 Spm improved photosynthesis

Changes in chl content can reflect the strength of photosynthesis, indicating the susceptibility of plants to drought stress (Ashraf and Harris, 2013). Many studies have demonstrated a gradual decrease in chl content under drought stress (Jeyaramrajaa et al., 2005; Li et al., 2024). In our study, drought stress caused a significant decrease in photosynthetic pigment contents in alfalfa. PA has been demonstrated that it can enter the chloroplast to scavenge excess ROS directly, thereby protecting the structural and functional integration of chloroplasts and slowing down the degradation of photosynthetic pigments (Nahar et al., 2015). In our study, exogenous Spm significantly increased the Chla, Chlb, and Car contents and Chla/Chlb and Car/Chl(a+b) ratios in alfalfa leaves. A study showed that Spd application greatly reduced chl losses in rice under aluminum stress (Jiang et al., 2021). This was similar to our findings. This is because Spm application slowed the decomposition of chl by regulating the activities of enzymes related to chl biosynthesis and decomposition (Zhou et al., 2016).

Stomatal control is recognized as a main physiological determinant for optimizing water use during drought stress, preventing excessive water loss under prolonged drought conditions (Nadeem et al., 2019). According to Farquhar and Sharkey (1982), the distinction between stomatal and nonstomatal limitations in photosynthesis is because when changes in the net photosynthetic rate and the intercellular carbon dioxide concentration are at equilibrium, the decrease in the net photosynthetic rate is determined by stomatal factors, whereas when the changes are in the opposite direction, the decrease in the net photosynthetic rate is determined by nonstomatal factors. In our study, drought stress decreased *P_n_* and *g_s_* and increased *C_i_*, suggesting that nonstomatal restriction is the main cause of the decrease in *P_n_* in G3 alfalfa plants under drought stress. Relatively drought-resistant cultivars maintained the dominant role of stomatal restriction under more severe drought stress conditions, which may partly explain the sensitivity of G3 to water stress. Exogenous Spm alleviated the decreases in *P_n_*, *g_s_* and *T_r_* and inhibited the increase in *C_i_* in alfalfa leaves under drought stress, which effectively alleviated nonstomatal limitations. This was consistent with the result that exogenous Spd alleviated the non-stomatal limitation of lettuce under high temperature stress (Yang et al., 2022). The probable reason may be that PAs enhanced plant photosynthesis by slowing down chl degradation and elevating Rubisco levels, thus alleviating the non-stomatal limitation of drought stress on alfalfa (Hassan et al., 2020).

Chl fluorescence reflects the absorbing, transferring and converting of light energy by leaves and performs an essential role in the study of photosynthesis and stress physiology (Chen et al., 2018). In our study, drought stress caused a decrease in the *Fv/Fm* ratio, *ΦPSII*, *qP* and *ETR* values of alfalfa leaves, which indicated a reduction in the photosynthetic potential of the *PSII* reaction center, a reduction in the efficiency of capturing excitation energy, and a reduction in the efficiency of the leaves in converting absorbed light energy into chemical energy, and that alfalfa was unable to efficiently utilize the absorbed light energy and was susceptible to photoinhibition (Piao et al., 2022). Exogenous Spm significantly slowed the reductions in the *Fv/Fm*, *ETR*, *qP* and *ФPSII* of alfalfa leaves under drought stress. It has been revealed that PA stimulates ATP synthesis, causes thylakoids accumulation, and improves the linear electron flow (LEF) rate and photochemical efficiency of *PSII* (Li et al., 2018). This may be the reason why alfalfa can maintain a relatively high level of *PSII* photochemical efficiency after applying Spm under drought stress. In addition, our results showed that the application of Spm significantly increased the *qN* of alfalfa leaves under drought stress, which indicated that Spm could improve the degree of nonphotochemical quenching and increase the energy dissipation of alfalfa leaves under drought stress, thus effectively mitigating damage to the photosynthetic apparatus. The above results showed that exogenous Spm could improve chl fluorescence parameters of alfalfa leaves under drought stress. These were similar to the results of exogenous Spd to increase *Fv/Fm*, *ΦPSII*, and *ETR* values in citrus (Chao et al., 2022) and lettuce (He et al., 2022) seedlings under high temperature stress.

### 4.3 Spm enhanced the antioxidant capacity

A reduction in *PSII* activity in plants under drought stress leads to an increase in excitation energy, causing an excess amount of energy and the production of excess ROS such as O_2_^−^·and H_2_O_2_, which can cause oxidative damage in plants (Sami et al., 2020). In response to damage caused by drought stress, many plants promote the synthesis of related enzymes such as SOD, POD, CAT, and APX and nonenzymatic antioxidants such as AsA and GSH to reduce oxidative damage, thereby alleviating drought stress damage to plants (Chakhchar et al., 2016; Kosar et al., 2021). In our study, drought stress induced O_2_^−^· and H_2_O_2_ accumulation and increased the MDA content, triggering membrane lipid peroxidation, which resulted in structural and functional damage to alfalfa membranes. After the application of Spm under drought stress, the O_2_^−^·, H_2_O_2_ and MDA contents of alfalfa leaves decreased, effectively alleviating the damage to cell membranes caused by drought stress. The observed results were attributed to enhanced SOD, CAT and POD activities and the AsA-GSH cycle. Previous studies also showed that exogenous Spm increased SOD, POD and CAT activities in winterberry plants exposed to drought stress (Xie et al., 2023), and exogenous Spd enhanced the ASA-GSH cycle in lettuce seedlings under high temperature stress (Li et al., 2020). These results were similar to our findings. The AsA-GSH cycle is a major system for scavenging ROS under abiotic stresses (Alzahrani et al., 2018; Raja et al., 2022). Empirical studies have demonstrated that upregulating or overexpressing AsA-GSH pathway enzymes and increasing AsA and GSH levels could reduce ROS levels, which could improve plant tolerance to abiotic stresses (Wang et al., 2022). High levels of APX can enhance drought tolerance in plants because APX utilizes AsA as an electron donor to reduce H_2_O_2_ to H_2_O (Anjum et al., 2016; Xu et al., 2022). In our study, drought stress did not significantly affect APX activity in G3 alfalfa leaves, but the application of Spm under drought stress increased APX enzyme activity in G3 alfalfa leaves, which partially explains the reduction in H_2_O_2_ after Spm application. At the same time, we observed that exogenous Spm application increased the AsA content and decreased the DHA content, which may be the result of exogenous Spm enhancing the conversion efficiency of DHA to ASA. GSH plays an important role in the ROS scavenging system, and GSH is oxidized to GSSG after H_2_O_2_ is scavenged. The GR activity of alfalfa decreased dramatically under drought stress, but exogenous Spm application increased the GR enzyme activity in G3 alfalfa leaves. At the same time, the GSH content in the leaves increased, and the GSSG content decreased. This is because GR can reduce GSSG to GSH, which is essential for maintaining a reduced state of GSH. Furthermore, the AsA/DHA and GSH/GSSG ratios can indicate dynamic changes in the cell redox state (Wang et al., 2011). Our study showed that exogenous Spm application increased the AsA/DHA and GSH/GSSG ratio of alfalfa under drought stress, suggesting that Spm effectively enhanced the ROS system, which further reduced the O_2_^−^·, H_2_O_2_, and MDA contents and attenuated the oxidative damage of alfalfa caused by drought stress. The possible mechanism by which Spm alleviates oxidative stress in stressed plants by regulating the antioxidant system, as well as altering ROS production and redox status, is that Spm may inhibit the autoxidation of metals, which reduces the supply of electrons required for ROS production and thus reduces ROS production (Shi et al., 2010). Moreover, Spm may enhance antioxidant activities by increasing nucleoside diphosphate kinase protein abundance (Shi et al., 2013a). In addition, Spm has been reported to act as part of the antioxidant system under stress conditions (Hasan et al., 2021). However, further studies are needed to decipher the specific mechanism by which Spm reduces the oxidative stress of plant cells subjected to drought.

### 4.4 Spm improved osmoregulation

The most essential impact of drought stress on plants is dehydration. In response to drought stress, plants accumulate a large amount of osmoregulatory substances to reduce their cellular osmotic potential and increase their cellular water uptake to resist damage caused by drought stress (Song et al., 2024). SS, SP and Pro are three important osmotic regulatory substances in plants. The results of this study showed that the SS, SP and Pro contents in alfalfa leaves significantly increased under drought stress. Exogenous Spm further increased the SS and Pro contents of alfalfa leaves under drought stress. A study indicated that exogenous Spd promoted the accumulation of Pro in *G. gandavensis* plants by up-regulating the expression of the 1-pyrroline-5-carboxylate synthetase (P5CS) gene, thereby enhancing the adaptation of *G. gandavensis* plants to salt stress (Qian et al., 2021). Therefore, we hypothesized that exogenous Spm application might promote Pro synthesis by enhancing the expression of the P5CS gene in alfalfa under drought stress. In addition, since PA and Pro biosynthesis share several substrates, the application of exogenous polyamines can modulate endogenous polyamine levels, thus providing more substrates for Pro biosynthesis (Shi et al., 2013b). It was reported that exogenous Spd induced an increase in the SS content of *Leymus chinensis* seeds under salt-alkali stress (Hongna et al., 2021). This was consistent with our results. This may be because several proteins involved in the photosystem of the calvin cycle, glycolysis and the gluconeogenesis/glyoxylate cycle are usually regulated by PAs, which leads to an increase in sugar content (Shi et al., 2013a). One study demonstrated that exogenous Spm application increased the SP content, thereby mitigating the adverse effects of a water deficit on safflower (Khosrowshahi et al., 2020). However, another study revealed that continuous spraying of Spd for 6 days had no significant effect on the accumulation of the SP content in lettuce under high-temperature stress, but continuous spraying of Spd for 8 days resulted in a significant increase in the SP content (Huang et al., 2023). Our results showed that exogenous Spm application did not significantly affect the SP content of alfalfa leaves under drought stress. This may be caused by the short processing time of the exogenous Spm application. This result also suggested that Pro and SS levels are more sensitive to hydration changes than are SP levels.

### 4.5 Integrated effects of Spm on physiological parameters

The PCA results showed that the application of Spm did not significantly affect the growth condition of alfalfa under normal water conditions, but the application of Spm under drought stress significantly increased the drought tolerance of alfalfa. It was previously shown that Spm reduced oxidative damage in tomato induced by combined salt and paraquat stresses, but Spm had no significant effect on tomato plants under non-stress condition (Pascual et al., 2023). This result was consistent with our findings. RDA results indicated that the addition of optimum concentration of Spm to alfalfa exposed to drought stress significantly affected CAT activity. This may be because CAT has been identified as a target enzyme for PA-induced S-nitrosylation. It was previously shown that the S-nitrosylation of CAT in citrus plants exposed to NaCl and PAs was accompanied by enhanced CAT activity compared to that in plants treated with NaCl alone (Tanou et al., 2014). Therefore, we hypothesized that exogenous Spm application contributes to the S-nitrosylation of CAT in alfalfa under drought stress, thereby dramatically increasing its activity. However, the mechanism on how s-nitrosylation enhances CAT activity is unclear and needs to be further investigated. In addition, the above results demonstrated that the addition of an optimal Spm concentration under drought stress could improve the drought tolerance of alfalfa by enhancing the ASA-GSH cycle. Similarly, exogenous Spd has been reported to alleviate oxidative damage caused by low-temperature stress in mung bean seedlings by enhancing the AsA-GSH cycle (Nahar et al., 2015). The FDA results indicated that the application of an optimum concentration of Spm under drought stress significantly increased the CAT activity, decreased the DHA content and increased the energy available for photochemical electron transfer in alfalfa, thus decreasing the production of H_2_O_2_. The coefficient of CAT activity is the largest among the four variables that make up the discriminant function. This suggested that CAT activity was the most important indicator for distinguishing drought stress treatment from drought stress with 1 mM Spm treatment. This was consistent with the results of our RDA results.

### 4.6 Spm enhanced TCA cycle

The type and content of metabolites change in response to different stimuli, leading to phenotypic changes. Metabolomic analysis revealed that the basic metabolic pathways involved in TCA cycling and arginine and Pro metabolism were altered in alfalfa leaves after the addition of Spm under drought stress. The maintenance of the accumulation of TCA cycle intermediates is an important regulatory mechanism for improving the drought tolerance of plants (Li et al., 2019). The accumulation of citric acid and alpha-ketoglutaric acid was inhibited by drought stress, whereas the addition of Spm increased the accumulation of oxobutanedioic acid, alpha-ketoglutaric acid, fumaric acid, citric acid and malic acid. This indicated that Spm regulated drought tolerance in alfalfa in association with better maintenance of the TCA cycle for energy production. Similarly, the mechanisms of salt resistance in soybean (Li et al., 2017) and drought resistance in garden asparagus (Zhang et al., 2024) were largely dependent on an increase in TCA cycle.

### 4.7 Spm increased ABA content

Interestingly, we observed a significant increase in ABA content after the application of exogenous Spm under drought stress. Tajti et al. (2019) reported that ABA accumulates in Spd-treated plants. This may be achieved by up-regulation of ABA synthesis gene expression by Spm (Marco et al., 2019). ABA plays a major role as a signaling molecule in response to drought stress (Sah et al., 2016). Accumulation of ABA induces stomatal closure, prevents leaf water loss, and alters the expression levels of genes involved in the stress response and biosynthesis of osmoregulatory substances, thereby helping to restore homeostasis to damaged cells (GusChina et al., 2002; Yoshida et al., 2003; Fujita et al., 2011). Previous studies have shown that ABA can induce the expression of plant antioxidant protective enzyme-encoding genes, such as the *Cu/Zn-SOD*, *Mn-SOD*, and *Fe-SOD* genes, as well as CAT-related genes (Guan and Scandalios, 1998; Kaminaka et al., 1999). In addition, ABA treatment increased the content of nonenzymatic antioxidants such as ASA, GSH and Car. Our physiological data showed that exogenous Spm application enhanced the antioxidant protection system, including enzymatic and nonenzymatic systems, in alfalfa under drought stress, possibly through the upregulation of ABA. It has been shown that ABA improves photochemical efficiency and low-temperature resistance in tomato by regulating chl synthesis and iron accumulation (Kang et al., 2023). A further study showed that the application of exogenous ABA mitigated the effects of abiotic stresses through an ABA-dependent signaling pathway related to the function of biological photosynthesis (Habibpourmehraban et al., 2023). Therefore, we hypothesized that exogenous spm enhanced photosynthesis in alfalfa under drought stress partly due to the accumulation of ABA.

### 4.8 Spm improved arginine and Pro metabolism

Arginine and Pro metabolism are vital not only for nitrogen assimilation, signal transduction and other physiological and biochemical processes in plants but also for osmotic regulation in plants under drought stress (Shi and Chan, 2013; Zhang et al., 2013a; Du et al., 2021). Metabolomic results showed that exogenous Spm resulted in significant accumulation of endogenous Spm in alfalfa under drought stress. This result was partly due to the direct uptake of exogenous Spm by the plant (Fujita and Shinozaki, 2015) and partly due to the stimulation of ABA synthesis by Spm, and ABA induced the expression of the Spm synthase gene, which led to Spm accumulation. It has been reported that the induction of the PA synthase genes *ADC2*, *SPDS1*, and *SPMS* in *Arabidopsis* by drought stress is an ABA-dependent response, as the upregulation of these genes was not observed in ABA-deficient (aba2) and insensitive (abi1) mutants (Alcázar et al., 2006). Endogenous Spm has been reported to regulate potassium channels and guard cells to control water loss by optimizing stomatal opening and closing (Agurla et al., 2018). In addition, many studies reported that high levels of endogenous Spm are beneficial in enhancing antioxidant enzyme activities in plants under stress conditions (Amini et al., 2021; Wang et al., 2023). In the metabolomic study, we also observed a further increase in the Pro content with the addition of Spm under drought stress, which is consistent with our physiological results. The large accumulation of Pro may be explained by the fact that ABA enhanced the expression of Pro-synthesizing enzyme-encoding genes (*P5CS* and *P5CR*) and suppressed the expression of Pro-degrading enzyme-encoding genes (*ProDH*) (Liu et al., 2022). Moreover, correlation analysis revealed a positive correlation between ABA and Pro contents (*p*<0.01).

### 4.9 Spm reduced MetSO accumulation

Met, an amino acid required for the synthesis of Spm, was significantly upregulated under drought stress, possibly because of the blocked conversion of Met to Spm under drought stress. Since Met is one of the most highly oxidized amino acids, under oxidative stress, it will quickly be converted to MetSO. MetSO can be converted to Met by methionine sulfoxide reductase (Msr), a process known to be crucial in the protection of cells against oxidative damage (Davies, 2005). A previous study reported NaCl-induced expression of genes of *OsMSRA4*, *OsMSRA5*, *OsMSRB3*, *OsMSRB5*, *OsMSRB1.1* and in rice roots, which was triggered by ABA accumulation under NaCl stress (Hsu and Lee, 2018). Under drought stress, MetSO accumulates in large quantities, resulting in severe oxidative damage to leaves, but the addition of Spm significantly reduces the accumulation of MetSO, which may be attributed to the fact that Spm maintains the expression of the Msr gene through the accumulation of ABA; however, further studies are needed.

## 5 Conclusion

**Figure 14:**
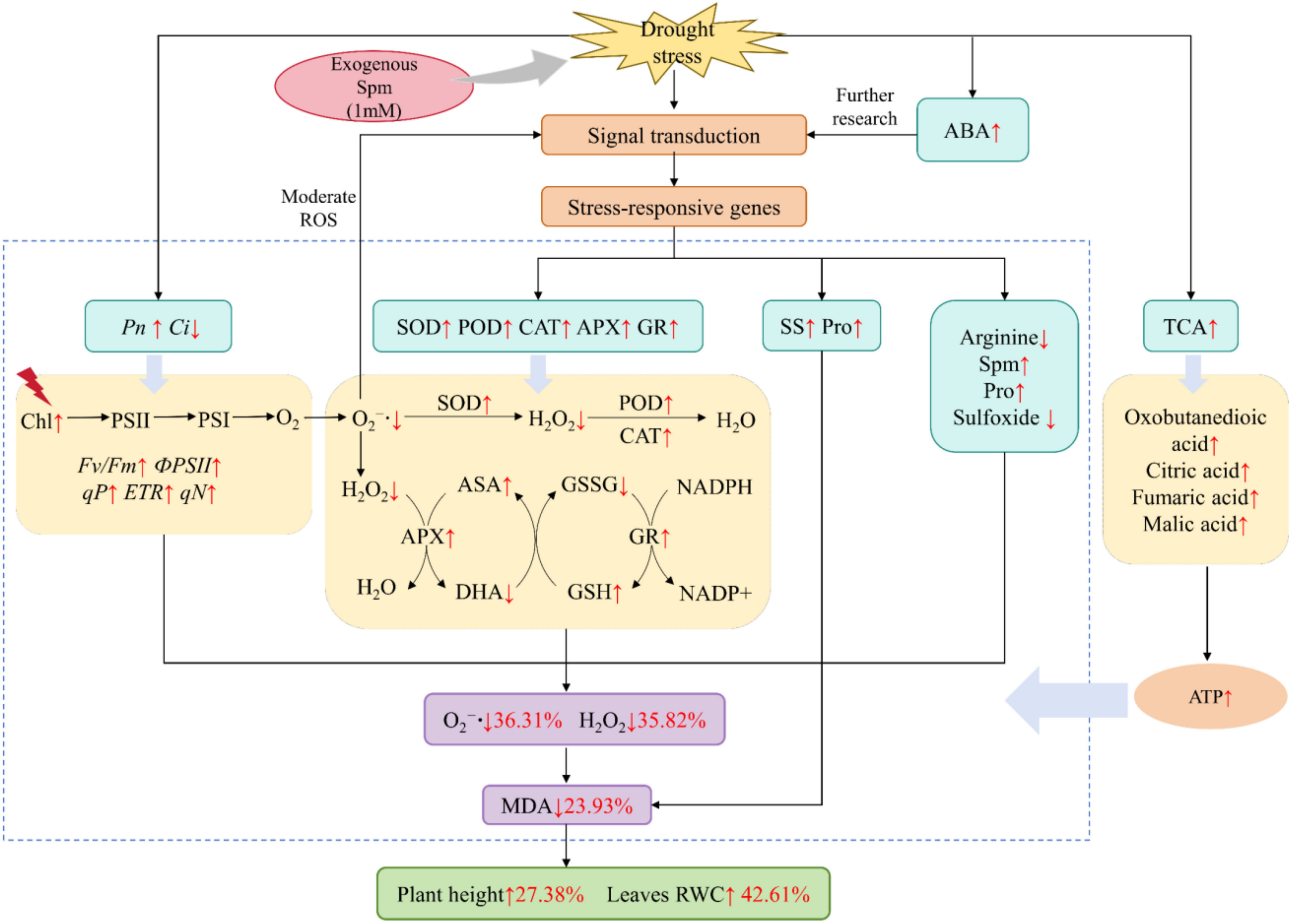
Physiological and metabolic mechanisms by which exogenous Spm enhances drought resistance in G3 alfalfa under drought stress. “↑” indicates a significant increase; “↓” indicates a significant decrease. The numbers following “↑” or “↓” represent the rates of increase or decrease, respectively.

In summary, at the physiological level, exogenous Spm alleviated non-stomatal limitation, increased photosynthetic rate, enhanced antioxidant enzyme activity, non-enzymatic antioxidant content, osmoregulatory compound content, and reduced ROS and MDA accumulation in alfalfa under drought stress, which in turn enhanced drought resistance in alfalfa. At the metabolic level, exogenous Spm enhanced drought tolerance in alfalfa through enhanced arginine and Pro metabolism and TCA cycle. In addition, metabolomics revealed that exogenous Spm increased the accumulation of the signaling substance ABA; therefore, we hypothesized that exogenous Spm application enhances drought resistance in alfalfa through the ABA signaling pathway, but this hypothesis requires further research (**Figure 14**). These findings can provide specific directions for further understanding the mechanism by which Spm promotes drought resistance in plants and provides a foundation for the practical application of Spm in the future.

## Data Availability

The data presented in the study are deposited in the OMIX repository, accession number OMIX007423.
